# Correction to “The impact of COVID‐19 on hospital‐based workers influenza vaccination uptake: A two‐year retrospective cohort study”Albanesi B, Clari M, Gonella S, et al. The impact of COVID‐19 on hospital‐based workers influenza vaccination uptake: a two‐year retrospective cohort study. J Occup Health. 2022;64:e12376. doi:10.1002/1348‐9585.12376

**DOI:** 10.1002/1348-9585.12394

**Published:** 2023-03-07

In the Acknowledgments section, “This study did not receive funds, grant, or other support.” was incorrect. This should have read “This research was financially supported by Fondazione CRT‐Turin, Italy [grant number 73537].”

We apologize for this error.

